# Recurrence of Primary Synovial Chondromatosis (Reichel's Syndrome) in the Ankle Joint following Surgical Excision

**DOI:** 10.1155/2021/9922684

**Published:** 2021-09-06

**Authors:** Thomas Gatt, Mark Portelli

**Affiliations:** Department of Orthopaedics and Trauma, Mater Dei Hospital, Malta

## Abstract

Primary synovial chondromatosis, or Reichel's syndrome, is a rare benign tumour arising from the synovial lining of a joint. We present the case of a 25-year-old male with Reichel's syndrome of the ankle, with subsequent recurrence following open retrieval of loose bodies. The initial presentation was of lateral malleolus discomfort which limited moderately strenuous exercise. Clinical examination showed a mild effusion and pain on extremes of movement. Imaging confirmed the presence of multiple loose bodies within the anterior and anterolateral recesses of the ankle. Open removal of 27 loose bodies from the joint was performed, with good postoperative recovery. He represented with pain 9 months later, with imaging of the ankle showing reaccumulation of loose bodies to a lesser extent. A trial of conservative management was opted for. Reichel's syndrome confined to the ankle is an exceedingly rare diagnosis, with few cases reported in the literature. This case saw the recurrence of the disease in a short time period despite successful surgery in the first instance. Management options to treat recurrence include repeat retrieval of foreign bodies, synovectomy, radiotherapy, or arthrodesis. While the prognosis is favourable, a low risk of malignant potential warrants adequate patient follow-up.

## 1. Introduction

Synovial chondromatosis (SC) is a rare benign tumour arising from the synovial lining of a joint [1]. This synovial metaplasia and subsequent proliferation leads to the formation of multiple cartilaginous loose bodies, which may calcify or ossify. The condition is classified as primary synovial chondromatosis, also known as Reichel's syndrome, when it is predominantly monoarticular and of unknown origin [2]. Secondary synovial chondromatosis occurs in association with degenerative changes in the joint, for instance, as a result of trauma or osteoarthritis. While the exact prevalence is unknown, it is estimated to effect around 1 in 100,000 individuals, with the majority of cases affecting large joints such as the knee and hip [3]. SC affecting the ankle joint is significantly rarer. In this case report, we present the case of a 25-year-old male with primary SC of the ankle, with subsequent recurrence following open retrieval of loose bodies.

## 2. Case Report

A 25-year-old male student presented with persistent discomfort over the lateral malleolus following an uncomplicated twisting inversion injury of the ankle 3 months prior. The pain was responding to bandage compression and simple analgesia, however was limiting moderately strenuous exercise. On examination, there was full range of movement at the ankle in all directions, with discomfort on maximal dorsiflexion and eversion. A mild effusion of the ankle joint was appreciated compared to the contralateral side. No ankle deformities or overlying skin changes were appreciated. The patient was previously healthy, on no regular medication. He smokes and drinks only socially. Routine blood investigations taken recently had been unremarkable.

**Figure 1:**
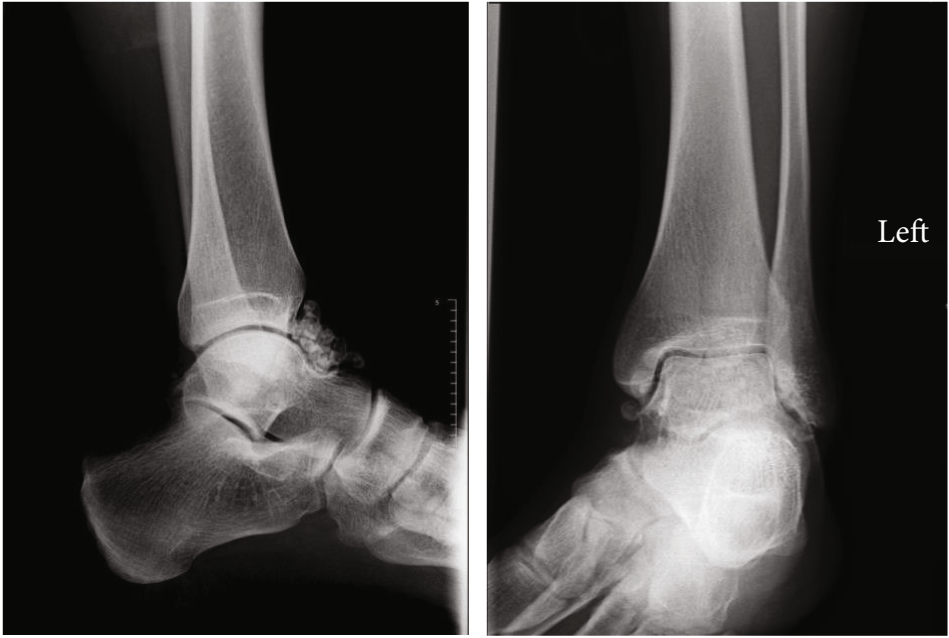
Lateral and mortise views of the left ankle joint on presentation.

An x-ray was performed on first presentation which demonstrated multiple globular calcified loose bodies in the anterior aspect of the joint, in keeping with suspected synovial chondromatosis (Figure 1). No fracture or joint deterioration was noted.

**Figure 2:**
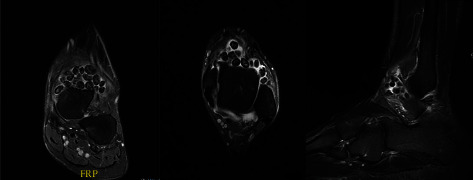
MRI demonstrating coronal, axial, and sagittal views of the ankle.

Magnetic resonance imaging of the ankle joint was performed to better visualize the lesions (Figure 2).

This confirmed the presence of multiple variably sized loose bodies within the ankle, primarily in the anterior and anterolateral recesses. The lesions were of indeterminate signal intensity on T1 and proton dense fat saturated images with a thin sclerotic rim. The largest loose body was 12 mm. The ankle and subtalar articular surfaces were normal with no evidence of osteochondral lesions or tarsal coalition. The visualized bones and ligaments were unremarkable.

**Figure 3:**
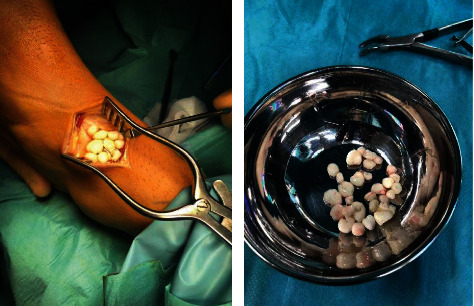
Intraoperative images of the ankle joint and the total amount of loose bodies removed.

Open removal of 27 loose bodies from the anterior gutter was performed, with the larger bodies sent for analysis (Figure 3). An open approach, as opposed to arthroscopic, was opted for in view of the large number of bodies and to visually ensure complete removal. The procedure was successful having obtained complete clearance with no intraoperative complications. The patient was discharged home on the same day with simple analgesia. He was advised to keep the limb elevated, allowed full weight bearing with crutches, and referred for physiotherapy. A postoperative review at two weeks showed no interval complications, good functional outcome, and benign histology. No further imaging or arthroscopy was deemed necessary as the patient had improved back to his premorbid state and was completely pain free at a three-month follow up.

**Figure 4:**
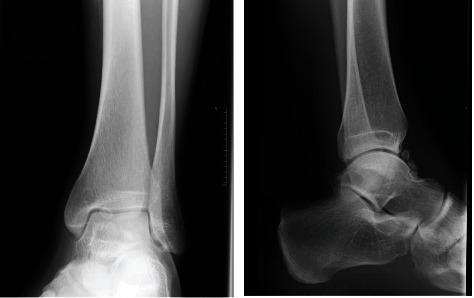
Mortise and lateral views of the ankle, 9 months after surgery, showing the reaccumalation of loose bodies.

The patient represented 9 months later, as he was once again experiencing ankle discomfort for the past two weeks. A repeat x-ray of the affected joint showed the reaccumulation of loose bodies, however to a much lesser extent (Figure 4). Once again, no fractures or joint abnormalities were detected. The management options were discussed, and the patient agreed to opt for a trial of conservative management, until the symptoms worsen. One year later, at the time of writing, his condition is clinically stable.

## 3. Discussion

Primary synovial chondromatosis (SC), or Reichel's syndrome, most commonly affects adults with a typical age presentation of 30 to 50 years and a predisposition towards males [1]. Radiologically, the condition differs from that of secondary SC since the loose bodies are typically more numerous and similar in size [4]. In our case, although the presentation followed an inversion injury, we feel that this was incidental in nature and not related to the development of SC. This assumption is based on the number and morphology of the loose bodies retrieved, as well as the preservation of normal joint anatomy on subsequent imaging.

While SC is typically a benign condition, there have been reports of malignant potential in the form of synovial osteochondroma, at an estimated rate of 5% [4, 5]. Distinguishing malignancy from SC is difficult even at a histological level, and clinical and radiological follow-up is advised [6]. Rapid clinical progression, bony involvement on imaging, or early and multiple recurrences may point towards malignant disease. Detailed imaging such as MRI of the joint is essential to rule out skeletal infiltration and aid in preoperative planning.

There is a scarcity in the literature of case series demonstrating SC confined solely to the ankle; however, the prognosis tends to be favourable [7–9]. Patients typically report good postoperative functional range of movement and improved pain scores. However, the recurrence rate after surgery is reported to range from 3 to 24%, with a higher rate of 35% described in a study by Murphey et al. and Galat et al. [4, 7] Unlike many case reports, the largest case series of foot SC had an average follow-up rate of 9.5 years, presumably contributing to this. In our case, despite complete removal of loose bodies via an open approach, symptoms began to recur relatively early within 9 months, albeit to a much lesser extent. Guidance in the literature on the optimum management of recurrence is scarce.

The management options for primary SC, as well as disease recurrence, are limited. Conservative management has been documented for asymptomatic patients or those not experiencing bothersome symptoms [10]. Repeat retrieval of loose bodies may be carried out and combined with synovectomy if this has not been done in the first instance. Synovectomy is indicated in cases where active synovitis is demonstrated [8]. While it provides pain-free return of function, comparative studies in the past have found no difference vis-a-vis recurrence rates when compared to open retrieval alone in initial surgery [11]. Meanwhile reports of an arthroscopic approach compared to open approach showed a superior recovery time in some studies [12, 13]. The use of hydrogen peroxide wash intraoperatively by Saxena and St. Louis is another technique claimed to reduce recurrence [14]. Apart from surgery, a novel approach of radiotherapy was described for the use of refractory SC by Chong et al. to good effect, however has yet to gain popularity [15]. The definitive treatment for recurrences remains arthrodesis. Despite this, Church et al. have reported an isolated case where recurrence despite arthrodesis is still possible [16].

## 4. Conclusion

Primary synovial chondromatosis confined to the ankle is an exceedingly rare diagnosis, with only a low volume of cases reported in the literature. Our case saw the recurrence of disease in a relatively short period despite complete removal of loose bodies in the first instance. Management options to treat recurrence include repeat open or arthroscopic retrieval of foreign bodies, synovectomy, radiotherapy, or arthrodesis. While the prognosis is favourable, a low probability of malignant potential should not be overlooked and thus patients warrant adequate follow-up.

## Data Availability

The data used to support the findings of this study are included within the article.
